# Vitamin B_12_ deficiency with combined hematological and neuropsychiatric derangements: a case report

**DOI:** 10.1186/1752-1947-8-277

**Published:** 2014-08-15

**Authors:** Luke Rannelli, Rita Watterson, Rupang Pandya, Alexander A Leung

**Affiliations:** 1 Department of Medicine, University of Calgary, Room 933, 9th Floor, North Tower, 1403 29 Street NW, Calgary, AB T2N 2T9, Canada; 2 Department of Psychiatry, Foothills Medical Centre, University of Calgary, Special Services Building, 1403 29 Street NW, Calgary, AB T2N 2T9, Canada; 3 Department of Psychiatry, University of Calgary, 1926-3500 26 Avenue NE, Calgary, AB T1Y 6J4, Canada; 4 Department of Medicine, University of Calgary, 313-4935 40 Avenue NW, Calgary, AB T3A 2N1, Canada

**Keywords:** Megaloblastic mania, Neuropsychiatric, Pancytopenia, Pernicious anemia, Vitamin B_12_ deficiency

## Abstract

**Introduction:**

Although vitamin B_12_ deficiency is a well-known cause of hematological and neuropsychiatric illness, the presentation of combined severe pancytopenia, demyelination and prominent psychiatric impairment is rare.

**Case presentation:**

We present a case of a previously healthy 55-year-old East African man with severe vitamin B_12_ deficiency (serum vitamin B_12_ 22pmol/L) secondary to pernicious anemia. He had a severe hypoproliferative megaloblastic anemia with hemolysis (hemoglobin 61g/L, mean corpuscular volume 99fL, reticulocytes 0.8%, haptoglobin undetectable), leukopenia (2.7×10^9^/L), thrombocytopenia (96×10^9^/L), ataxia with central demyelination, and megaloblastic madness. The patient’s anemia, myelopathy and psychiatric condition responded well to parenteral vitamin B_12_ replacement therapy, with significant improvement seen within weeks.

**Conclusion:**

Hematological manifestations of vitamin B_12_ deficiency are typically inversely correlated with the presence and severity of neuropsychiatric impairment. Although uncommon, a presentation with severe hematological and neuropsychiatric disease can occur, as illustrated by this case. Its presence may help guide diagnosis as well as provide clinically important prognostic information.

## Introduction

Vitamin B_12_ is essential for normal blood synthesis and neurological function. Vitamin B_12_ deficiency may cause megaloblastic anemia, subacute combined degeneration of the cord and psychiatric illness (“megaloblastic madness”). Notably, the degree of bone marrow suppression is typically inversely related to both the presence and severity of neurological involvement. As such, the coexistence of significant anemia and neurological deficits is thought to be rare. We describe an unusual case of a patient with severe vitamin B_12_ deficiency together with profound hematological derangements and florid neuropsychiatric impairment.

## Case presentation

A 55-year-old man of East African descent presented to our community hospital with a history of repeated falls, postural dizziness, progressive fatigue, generalized weakness and 30-lb weight loss over the course of three to six months. He is a vegetarian. His collateral history obtained from family members confirmed a significant behavioral change over the past year, during which they observed irritability and emotional lability, as well as difficulty with self-care, grooming and personal hygiene. He was high-functioning before the onset of his present illness. During a review of systems, he denied any sensory deficits, visual impairment or taste perversion, and he also denied any history of bleeding, bruising or recent infection. His past medical history was unremarkable. He was not taking any medications at the time of presentation. The patient denied smoking, alcohol consumption or illicit drug use. He had no significant family history of any illness.

The initial examination in the emergency department revealed a poorly groomed, disheveled thin man who appeared older than his stated age. He was irritable, paranoid, delusional and highly circumstantial and had grandiose thoughts and impaired insight. He denied any hallucinations. The patient’s blood pressure was 96/50mmHg without postural change, his pulse was 92 beats/min and his respiratory rate was 16 breaths/min. He was afebrile. His neurological examination was limited because he was uncooperative, but he was observed to have an unstable, wide-based, ataxic gait as well as difficulty in performing rapid, alternating hand movements. His proprioception, vibration sense and light-touch sensory modalities appeared grossly preserved. His reflexes were symmetric and normal. His cardiovascular, respiratory, abdominal and dermatological examination results were unremarkable. Of note, he did not have evidence of splenomegaly, oral ulcers, nail changes or vitiligo. The patient was subsequently admitted to the hospital for further evaluation.

A complete blood count revealed hemoglobin 61g/L (mean corpuscular volume 99fL), thrombocytopenia 96×10^9^ and leukopenia 2.7×10^9^. The patient’s reticulocytes were decreased at 14×10^9^/L (0.8%). A peripheral blood smear demonstrated hypersegmented neutrophils and schistocytes. His serum vitamin B_12_ level was decreased at 22pmol/L (reference range 155pmol/L to 700pmol/L), his serum haptoglobin was undetectable and his serum lactate dehydrogenase was elevated at 1135U/L. The patient’s iron studies, folate, thyroid-stimulating hormone, liver enzymes, fibrinogen, serum protein electrophoresis, anti–tissue transglutaminase antibodies, hemoglobin A1c, coagulation panel, electrolytes and serum creatinine were all within normal limits. Viral studies for parvovirus B19, cytomegalovirus and human immunodeficiency virus were unremarkable. However, anti–intrinsic factor antibodies were detected. His blood type was B+. Of note, all blood work was performed prior to commencing any medical treatment or blood transfusions.

**Figure 1:**
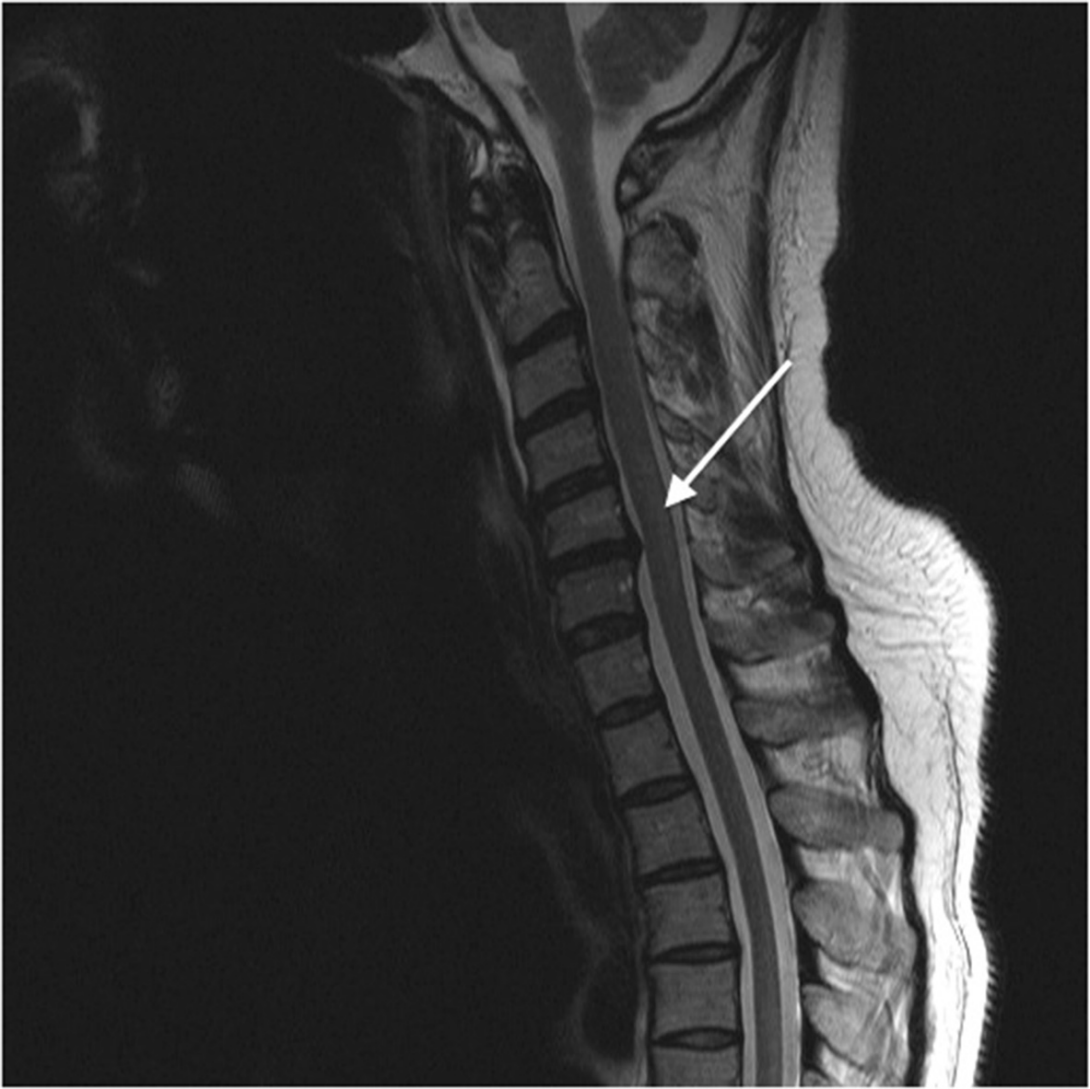
**Magnetic resonance imaging scan of the patient’s spine.** This sagittal view of the patient’s cervical and thoracic spine shows an area of hyperintensity (indicated by the arrow) along the dorsal column, consistent with demyelination due to vitamin B_12_ deficiency.

Computed tomography of the head revealed mild cerebral atrophy. Magnetic resonance imaging confirmed the presence of mild cerebral atrophy with increased signal intensity throughout the dorsal aspect of the cervical and thoracic spine, consistent with vitamin B_12_ myelopathy (Figure 1). Chest radiography and computed tomography of the abdomen and pelvis were unremarkable. The patient did not consent to undergo diagnostic endoscopy. The patient was also unwilling to undergo electromyography with nerve conduction studies.

The diagnosis of severe vitamin B_12_ deficiency secondary to pernicious anemia was established on the basis of an unequivocally low serum vitamin B_12_ level and the presence of anti–intrinsic factor antibodies. The patient’s long-standing vegetarian diet may also have been a contributor to his vitamin B_12_ deficiency. Other causes of pancytopenia which may be characterized by a hypoproliferative, megaloblastic, macrocytic anemia—such as folate deficiency, hypothyroidism, viral illness and exposure to potential offending medications—were ruled out. The patient’s formal neurological and psychiatric evaluations were also consistent with central demyelinating disease and megaloblastic madness with prominent depressive features, after potential confounding metabolic and anatomical causes were excluded.

Because of the profound symptomatic anemia at presentation, the patient was initially given a transfusion of 2U of packed red blood cells upon admission. He was then initiated on daily subcutaneous injections of vitamin B_12_ at 1000μg for one week, followed by weekly injections for the next four weeks, and he was subsequently prescribed monthly injections to be continued indefinitely. Within the first week of treatment, the patient’s pancytopenia had nearly resolved. His neuropsychiatric symptoms and cognition had also improved significantly by the time of hospital discharge. At follow-up three months later, the patient’s neuropsychiatric impairment had completely resolved. He denied any psychiatric disturbance, demonstrated a normal gait and had normal reflexes upon follow-up.

## Discussion

Vitamin B_12_ deficiency is common, with an estimated prevalence of 15% in patients >60 years of age. Although most cases are clinically subtle, vitamin B_12_ deficiency may sometimes present with florid disease [1,2]. When present, symptoms and signs can be broadly classified into hematological and neurological categories. Classically, vitamin B_12_ deficiency may give rise to megaloblastic anemia and sub-acute combined degeneration of the spinal cord with demyelination of the dorsal column, resulting in both motor and sensory deficits. Less commonly, hemolysis and pancytopenia may be detected in the blood work, and neurological sequelae of optic nerve atrophy, autonomic nervous system dysfunction, peripheral neuropathy, as well as psychiatric complications of emotional lability, mania, paranoia, delusions, amnesia and psychosis, may also be present [3].

Notably, the degree of megaloblastic anemia in a patient with vitamin B_12_ deficiency is inversely correlated with the presence and severity of neuropsychiatric symptoms at the time of presentation [3-6]. Severe anemia is rarely accompanied by any neurological symptoms or signs [5]. Likewise, hematocrit and mean corpuscular volume are commonly normal in patients with neuropsychiatric abnormalities [3,5]. The underlying reason for this relationship remains unclear. However, it is postulated that the mechanism responsible for the neurological dysfunction associated with vitamin B_12_ deficiency is altogether different from those responsible for the hematological sequelae. Vitamin B_12_ acts as a coenzyme for L-methylmalonyl-coenzyme A mutase and methionine synthetase. Accordingly, enzymatic defects resulting from vitamin B_12_ deficiency lead to accumulation of methylmalonic acid and homocysteine, which appear to be proportionally related to the severity of the associated neurological and psychiatric abnormalities [4]. In contrast, inadequate vitamin B_12_ results in ineffective DNA synthesis and impaired erythropoiesis, which account for the majority of the associated hematological derangements [3]. The underlying mechanisms responsible for the resulting megaloblastic anemia appear to be altogether different from the processes that produce the neurological sequelae of vitamin B_12_ deficiency.

Myelopathy and peripheral neuropathy collectively account for the vast majority of neurological dysfunction [6], with mental impairment representing approximately 15% of cases [4,6]. Among patients with neurological disease, anemia and macrocytosis are the most common hematological derangements detected, and, when they are present, anemia and macrocytosis are typically minor and subtle [4,6]. In contrast, pancytopenia, severe anemia (that is, <60g/L), hemolysis, leukopenia and thrombocytopenia are all documented to be rare [2,3,5].

The presence of anemia also carries important prognostic significance. Anemia detected at baseline is inversely related to the severity of neurological impairment at diagnosis. Even after adjusting for differences in age, sex, mean corpuscular volume, serum vitamin B_12_ level and disease duration, a lower hematocrit level remains an independent predictor of worse neurological disease [5]. It appears that the presence of anemia at baseline also favors a better neurological response to treatment and a more favorable long-term recovery [5].

## Conclusion

Pernicious anemia can present with severe hematologic and neuropsychiatric conditions concurrently, contrary to stated dogma. Transient, self-limited neurological exacerbations may sometimes occur during the first few weeks of treatment. However, appropriate therapy can lead to complete recovery in up to two-thirds of patients treated [2,3].

## Consent

Written informed consent was obtained from the patient for publication of this case report and any accompanying images. A copy of the written consent is available for review by the Editor-in-Chief of this journal.

## Competing interests

The authors declare that they have no competing interests.

## Authors’ contributions

LR, RW and AL were involved in drafting, writing and editing the manuscript. RP contributed to the writing and editing of the manuscript. All authors were directly involved in the care of the patient. All authors read and approved the final manuscript.
